# Benzyl Isothiocyanate Suppresses Pancreatic Tumor Angiogenesis and Invasion by Inhibiting HIF-α/VEGF/Rho-GTPases: Pivotal Role of STAT-3

**DOI:** 10.1371/journal.pone.0025799

**Published:** 2011-10-10

**Authors:** Srinivas Reddy Boreddy, Ravi P. Sahu, Sanjay K. Srivastava

**Affiliations:** Department of Biomedical Sciences and Cancer Biology Center, School of Pharmacy, Texas Tech University Health Sciences Center, Amarillo, Texas, United States of America; Texas A & M University, United States of America

## Abstract

Our previous studies have shown that benzyl isothiocyanate (BITC) suppresses pancreatic tumor growth by inhibiting STAT-3; however, the exact mechanism of tumor growth suppression was not clear. Here we evaluated the effects and mechanism of BITC on pancreatic tumor angiogenesis. Our results reveal that BITC significantly inhibits neovasularization on rat aorta and Chicken-Chorioallantoic membrane. Furthermore, BITC blocks the migration and invasion of BxPC-3 and PanC-1 pancreatic cancer cells in a dose dependant manner. Moreover, secretion of VEGF and MMP-2 in normoxic and hypoxic BxPC-3 and PanC-1 cells was significantly suppressed by BITC. Both VEGF and MMP-2 play a critical role in angiogenesis and metastasis. Our results reveal that BITC significantly suppresses the phosphorylation of VEGFR-2 (Tyr-1175), and expression of HIF-α. Rho-GTPases, which are regulated by VEGF play a crucial role in pancreatic cancer progression. BITC treatment reduced the expression of RhoC whereas up-regulated the expression of tumor suppressor RhoB. STAT-3 over-expression or IL-6 treatment significantly induced HIF-1α and VEGF expression; however, BITC substantially suppressed STAT-3 as well as STAT-3-induced HIF-1α and VEGF expression. Finally, *in vivo* tumor growth and matrigel-plug assay show reduced tumor growth and substantial reduction of hemoglobin content in the matrigel plugs and tumors of mice treated orally with 12 µmol BITC, indicating reduced tumor angiogenesis. Immunoblotting of BITC treated tumors show reduced expression of STAT-3 phosphorylation (Tyr-705), HIF-α, VEGFR-2, VEGF, MMP-2, CD31 and RhoC. Taken together, our results suggest that BITC suppresses pancreatic tumor growth by inhibiting tumor angiogenesis through STAT-3-dependant pathway.

## Introduction

The prognosis for patients with advanced pancreatic cancer remains poor with a median survival of only 6 months, making it the fourth leading cause of cancer-related deaths in both men and women [1], [2]. Minimal effect of conventional chemotherapy drugs, including gemcitabine, on patient survival rates underscores the need for new strategies to inhibit pancreatic tumor growth [3]. Pancreatic tumors can trigger substantial development of new blood vessels in a process called angiogenesis. This vascular development is a necessary component of solid tumor growth and progression [4]. Numerous reports have shown that disrupting tumor angiogenesis effectively inhibits tumor growth and metastasis [5], [6], [7].

Various stimuli such as hypoxia, inflammation, and mechanical stress and stretch are known to initiate angiogenesis [8]. Critical steps of angiogenesis including migration, invasion, and proliferation are mediated by complex signaling proteins such as hypoxia inducible factor (HIF-α), vascular endothelial growth factor (VEGF), and matrix metalloproteinases (MMPs) [8]. During hypoxia, HIF-α protein levels increase due to a decreased rate of ubiquitin-mediated degradation [9]. Up-regulation of the HIF-1 system is observed in many cancers and is caused by multiple genetic and environmental factors [10]. VEGF expression is regulated by HIF-1α-dependent and -independent mechanisms [11]. Recent studies have identified the signal transducer and activator of transcription 3 (STAT-3) as a direct transcriptional activator of VEGF and HIF-1α under hypoxia [12], [13]. Moreover, constitutive activation of STAT-3 occurs at a frequency of 50–90% in a broad range of human cancers, suggesting that STAT-3 activity contributes significantly to tumor VEGF overproduction [12]. Previous reports have shown that phosphorylated STAT-3 is associated with over-expression of VEGF and HIF-1α in human pancreatic tumors and that inhibition of STAT-3 causes significant reduction in tumor growth and vascularization [14]. Recent reports show that Rho-GTPases, which are downstream of VEGF signaling, play a vital role in all stages of cancer progression, including metastasis [15], [16]. Thus, the disruption of tumor angiogenesis resulting from the inhibition of STAT-3, HIF-α/VEGF/Rho-GTPases, and MMPs production represents a promising strategy for cancer therapy.

Case-controlled epidemiological studies continue to support the notion that consumption of cruciferous vegetables reduces the risk of pancreatic cancer [17], [18]. Benzyl isothiocyanate (BITC) present in cruciferous vegetables such as watercress and garden cress inhibits chemically-induced cancers in experimental animals [19], [20]. Previous studies, including those from our laboratory, have shown that BITC effectively suppresses the growth of human pancreatic cancer cells both *in vitro* and *in vivo* by causing apoptotic cell death through MAPK activation or NF-kB inhibition [21], [22], [23], [24]. Our previous studies have demonstrated that BITC suppresses pancreatic tumor growth by inhibiting STAT-3 [23]. We also have shown that normal human pancreatic epithelial cells were least affected by BITC treatment [22], [23], [24]. However, it still is not clear if BITC suppresses pancreatic tumor progression by inhibiting tumor angiogenesis. Therefore, this study evaluated the effect of BITC on pancreatic tumor angiogenesis and investigated the underlying molecular mechanism. We provide evidence that BITC dose-dependently inhibits the migration, invasion, and neovascularization of human pancreatic cancer and endothelial cells by targeting HIF1α, VEGF, MMP-2, and Rho-GTPases through the STAT-3 dependent pathway.

## Results

### Inhibition of neovascularization by BITC

**Figure 1:**
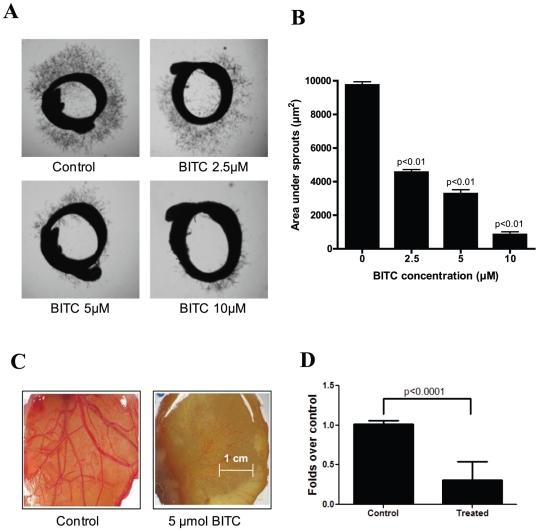
BITC inhibits angiogenesis *ex vivo*. **A**. BITC inhibits VEGF-induced vessel sprouting *ex vivo*. Aortic rings (1 mm) were harvested from Sprague-Dawley rats, immerged in matrigel, and treated with VEGF (20 ng/mL) in the absence or presence of BITC (0, 2.5 and 5 and 10 µM) for 4 days and photographed under microscope (4X). Representative photographs are presented. **B**. Quantitative analysis of aortic ring assay. Aortic ring sprouting was quantified by Image J software and presented as mean ± SD of triplicates. **C**. Inhibition of CAM angiogenesis by BITC. Eggs were incubated at 37°C for 3 days. A Whatman filter disc containing the test compound (BITC 5 µmol) was placed on the CAM of eggs (n = 10) through pre-opened window and further incubated. On day 9–12 of incubation, photographs were made after removing the filter discs. A representative photograph is presented. **D**. Blood vessels density was quantified by Image J software and represented as a bar diagram.

To address whether BITC inhibits angiogenesis, rat aortic rings embedded in matrigel were incubated with different concentrations of BITC. Aortic sprouting was initiated by treating the rings with 20 ng/mL VEGF. Treatment with 5 µM BITC reduced sprouting of new blood vessels by 67% as compared to control aortic rings (Fig. 1A). The area under the sprout outside the treated rings (845 µm^2^) was reduced by 91% as compared to control rings (9747 µm^2^) (Fig. 1B).

To confirm the observations made in rat aortic ring assay, we examined the effect of BITC on CAM angiogenesis. As shown in Fig. 1C, 5 µmol BITC treatment drastically suppressed new embryonic blood vessel growth in each egg as compared to control eggs. The number of newly formed blood vessels was suppressed by about 70% in BITC-treated CAMs as compared to control CAMs (Fig. 1C & D).

### BITC inhibits secretion of MMP-2 and VEGF from pancreatic tumor cells under normoxic and hypoxic conditions

**Figure 2:**
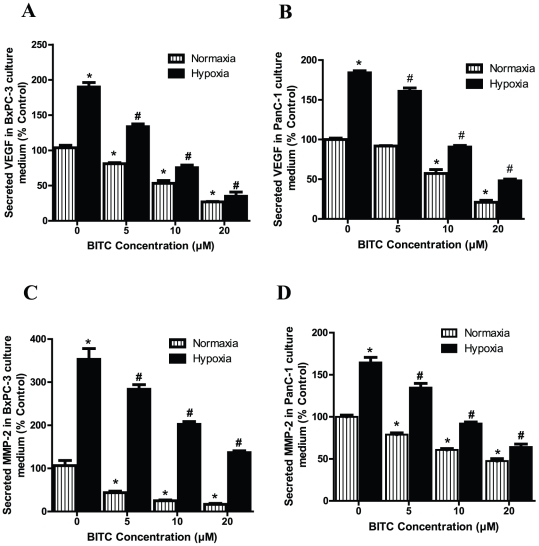
BITC inhibits secretion of proangiogenic factors from BxPC-3 and PanC-1 cells under normoxic and hypoxic conditions. Serum-starved BxPC-3 or PanC-1 cells were treated with various concentrations of BITC in a 96-well plate and incubated for 24 h. For hypoxia treatment, cells were exposed to 1% pO_2_ for 24 h. Culture supernatants were collected and assayed for MMP-2 or VEGF by ELISA kit -. **A–B**. BITC suppresses secretion of VEGF from BxPC-3 and PanC-1 cells. **C–D**. BITC blocks secretion of MMP-2. Values are mean ± SD of triplicates. *p<0.01 statistically significant when compared with normoxic controls. #p<0.01, statistically significant when compared with hypoxic controls.

In the tumor microenvironment, migration, invasion, and neovascularization are critically regulated by proangiogenic factors such as VEGF and MMP-2 secreted by tumor cells [25], [26]. Therefore, we next examined whether BITC suppresses the secretion of VEGF and MMP-2 from BxPC-3 and PanC-1 pancreatic tumor cells. BITC treatment significantly inhibited secreted levels of VEGF in both cell lines in a dose-dependent manner. Treatment with 10 µM BITC inhibited VEGF levels in the BxPC-3 cells by about 64% and in the PanC-1 cell by about 53% (Fig. 2A–2B). Similarly, 10 µM of BITC inhibited MMP-2 secretion in BxPC-3 cells by 79% and in PanC-1 cells by 60%. (Fig. 2C–2D).

Since we observed that BITC blocked the secretion of VEGF and MMP-2 in BxPC-3 and PanC-1 cells under normoxic conditions, we next sought to determine whether BITC causes a similar effect under hypoxic conditions. Our results show that VEGF secretion increased approximately 2 fold (Fig. 2A) and MMP-2 levels increased around 3.5 fold in hypoxic BxPC-3 cells, as compared to normoxic cells. These observations were consistent with previous reports showing increased secretion of VEGF and MMP-2 under hypoxic conditions [27]. In both BxPC-3 and PanC-1 cells, hypoxia-induced enhanced VEGF and MMP-2 secretion levels were substantially reduced by BITC in a dose-dependent manner (Fig. 2A–2D). For example, 10 µM BITC inhibited 70% and 51% of VEGF secretion and 60% and 44% of MMP-2 secretion by BxPC-3 and PanC-1 cells respectively, as compared to hypoxic control cells.

### BITC down-regulates HIF-α, VEGFR-2, MMP-2, and Rho-GTPases

**Figure 3:**
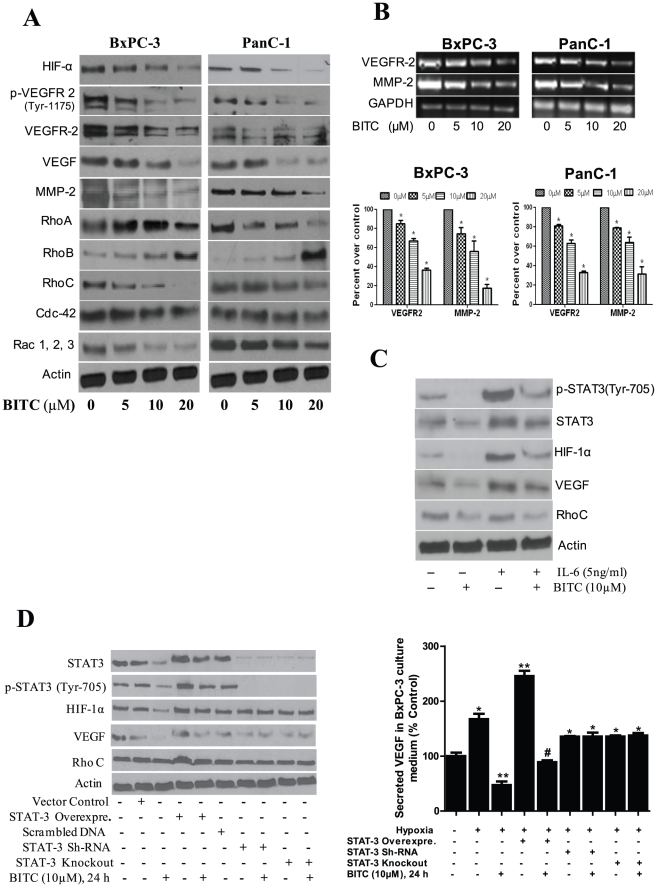
BITC suppresses proangiogenic proteins. **A**. BITC down-regulates expression of HIF-α, VEGFR-2, Rho-GTPase signaling molecules. BxPC-3 or PanC-1 cells were treated with various concentrations of BITC for 24 h and for HIF-α, cells were exposed to 1% O_2_ for 12 h. Whole cell lysates were prepared with urea-CHAPS buffer. Cell lysates were analyzed by SDS-PAGE followed by western blot. **B**. BITC down-regulates mRNA levels of VEGFR2 and MMP-2 in BxPC-3 and PanC-1 cells. Cells were treated with different concentrations of BITC and total RNA was isolated with Trizol. Total RNA was analyzed for the expression levels of VEGFR-2 and MMP-2 by RT-PCR. GAPDH was used as internal control. Quantitative analysis of mRNA expression levels were performed by Image J software and presented as bar diagram (lower panel) **C**. BITC inhibits HIF-1α and VEGF expression by inhibiting phosphorylation of STAT-3. 0.3×10^6^ cells were plated in 6-well plate and treated with 20 ng/mL IL-6 and 10 µM BITC for 24 h. Cells were analyzed for STAT-3 (Tyr 705), STAT-3, HIF-1α, VEGF, and RhoC expression by western blot. **D**. STAT-3 is required for BITC mediated inhibition of HIF-1α and VEGF expression. BxPC-3 cells were transfected with 2 µg of STAT-3α over-expressing plasmid and, in another experiment, STAT-3 was either transiently silenced or permanently knocked out by shRNA. Transfected cells were treated with or without 10 µM BITC for 24 h after 48 h of transfection. Cells were lysed and analyzed by western blot. Right panel showing secreted VEGF level was evaluated in STAT-3 over-expressing or silenced BxPC-3 cells by ELISA as described above. * Values of p<0.01 statistically significant when compared with normoxic controls. **p<0.01 statistically significant when compared with hypoxic controls. #p<0.01statistically significant when compared with STAT-3 over-expressing cells.

To explore the molecular mechanism of the antiangiogenic effects of BITC, BxPC-3 and PanC-1 cells were immunoblotted for various pro-angiogenic proteins. Our results indicate that BITC significantly reduced hypoxia-induced HIF-1α expression in both BxPC-3 and PanC-1 cells (Fig. 3A). Furthermore, our results reveal that BITC significantly down-regulated the expression and the phosphorylation (activation) of VEGFR-2 (Tyr-1175) as well as the expression of VEGF and MMP-2 in both BxPC-3 and PanC-1 cells (Fig. 3A). To examine whether down regulation of VEGFR-2 and/or MMP-2 expression by BITC was at the transcriptional level, mRNA was analyzed by RT-PCR. Our results reveal that mRNA levels of both VEGFR-2 and MMP-2 were reduced significantly in both the cell lines in response to treatment (Fig. 3B), indicating that BITC inhibits both VEGF and MMP-2 at the transcriptional level. To further delineate the mechanism, cell lysates were probed for VEGFR-2 downstream proteins such as Rho-GTPases, which play a critical role in cancer progression and metastasis. Our results demonstrate that the expression of RhoA, RhoC, Cdc-42, and Rac 1,2,3 were significantly reduced by BITC treatment, whereas RhoB expression levels were up-regulated in BxPC-3 and PanC-1 cells in a dose-dependent manner (Fig. 3A).

### BITC suppresses STAT-3 mediated induction of HIF-1α and VEGF expression

Previous reports have identified STAT-3 as a direct transcriptional activator of HIF-1α and VEGF during hypoxia [12], [13]. We therefore wanted to see whether inhibition of HIF-1α and VEGF by BITC was mediated through STAT-3. To address this question, BxPC-3 cells were treated with IL-6, which specifically activates STAT-3 by phosphorylation at Tyr-705, and determined HIF-1α and VEGF expression. Our results show that IL-6 drastically increased the expression of HIF-1α and VEGF in BxPC-3 cells. When these cells were treated with BITC, IL-6-induced HIF-1α and VEGF expression was substantially reduced (Fig. 3C). These results indicate that BITC inhibits HIF-1α and VEGF through STAT-3 dependent pathway.

### STAT-3 is obligatory for BITC mediated HIF-1α and VEGF inhibition

To further confirm the involvement of STAT-3 in BITC mediated HIF-1α and VEGF inhibition, STAT-3 was either transiently over-expressed or silenced in BxPC-3 cells. Our results show that over-expression of STAT-3 in BxPC-3 cells significantly increased the expression of HIF-1α and VEGF under hypoxia (Fig. 3D). On the other hand, silencing STAT-3 with shRNA in BxPC-3 cells showed a modest decrease in the expression of HIF-1α and VEGF as compared to control cells under hypoxia (Fig. 3D). Similar observations were made in STAT-3 stably knocked out BxPC-3 cells (Fig. 3D), indicating the pivotal role of STAT-3 in hypoxia-induced HIF-1α and VEGF expression. When STAT-3 over-expressing BxPC-3 cells were treated with BITC under hypoxia, the HIF-1α and VEGF levels were significantly reduced (Fig. 3D), confirming the role of STAT-3 in BITC-mediated HIF-1α and VEGF inhibition. Furthermore, in STAT-3 transiently silenced or permanently knocked out cells, BITC had little effect on HIF-1α and VEGF expression. Our results were further confirmed by VEGF ELISA studies in STAT-3 over-expressing or STAT-3 silenced BxPC-3 cells under hypoxia (Fig. 3D, right panel). These studies clearly establish the mechanistic role of STAT-3 in regulating HIF-1α and VEGF in our model and confirm that the inhibition of HIF-1α and VEGF by BITC was in fact mediated through STAT-3 inhibition.

### BITC inhibits migration and invasion of BxPC-3 cells

**Figure 4:**
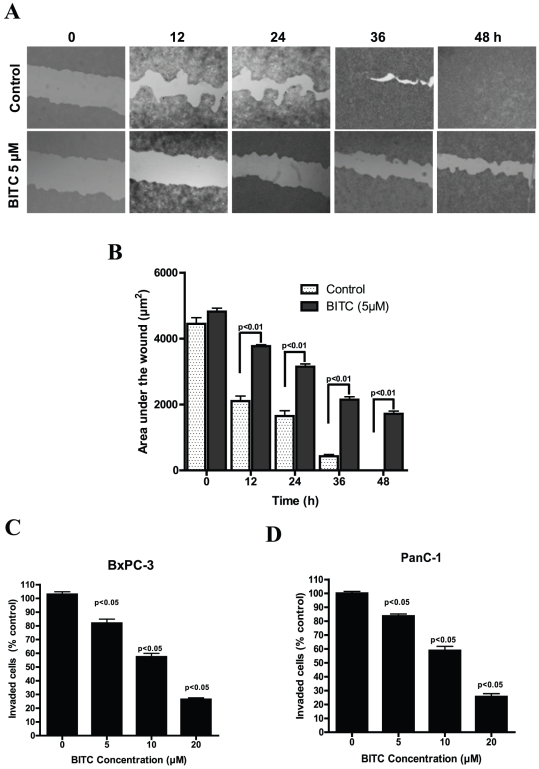
BITC inhibits migration and invasion of pancreatic cancer cells. **A**. BITC inhibits migration of BxPC-3 cells. BxPC-3 cells were plated, scratched with pipette tip, and incubated in the absence or presence of 5 µM BITC. Photomicrographs were made at regular intervals using inverted microscope. **B**. Quantitative representation of migration assay. Wound area in BITC-treated and control cells were quantified by Image J software and presented as mean ± SD of triplicates. p<0.01, statistically significant when compared to corresponding time points in controls cells. **C–D**. BITC inhibits the invasion of BxPC-3 and PanC-1 cells. Invasion assay was performed using Boyden's chamber. Results are presented as mean ± SD of triplicates. p<0.05, statistically significant when compared controls.

Since the migration of endothelial or epithelial cells is one of the key steps in angiogenesis and metastasis [8], we next explored the effect of BITC on the migration of BxPC-3 cells in a wound healing assay. Our results show that 5 µM BITC significantly blocked the migration of BxPC-3 cells (Fig. 4A). After 36 hours, about 90% of BxPC-3 cells migrated into the wounded area, whereas migration of BITC treated cells was around 55%, indicating a potential anti-migration effect of BITC (Fig. 4B). We next wanted to see whether BITC inhibits the invasion of BxPC-3 and PanC-1 cells. Treatment with 10 µM BITC inhibited around 50% of both BxPC-3 and PanC-1 cell invasion as compared to untreated cells (Fig. 4C–4D). These results suggest that BITC may prevent the entry of tumor cells into distal locations *in vivo* and thereby inhibit the metastasis of primary tumors.

### Effect of BITC on human endothelial (HUVEC) cells

**Figure 5:**
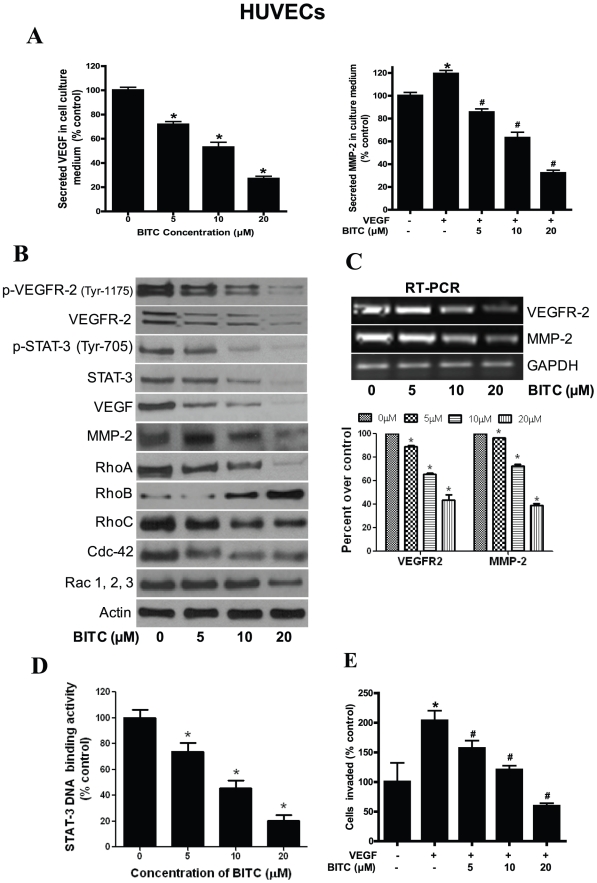
BITC inhibits angiogenesis in HUVECs. **A**. BITC inhibits the secretion of proangiogenic factors from HUVECs. Cells were plated, stimulated with VEGF, and treated with BITC for 24 h. Media was collected and assayed for MMP-2 and VEGF by ELISA kit. *p<0.01 statistically significant when compared with controls. #p<0.01 statistically significant when compared with VEGF-stimulated controls. **B**. Regulation of VEGF mediated signaling by BITC. HUVECs were treated with various concentrations of BITC and whole cell lysates were analyzed by western blot. **C**. BITC down-regulates VEGFR2 and MMP-2 mRNA in HUVECs. Total RNA from BITC-treated HUVECs was isolated with Trizol and analyzed for the expression levels of VEGFR-2 and MMP-2 by RT-PCR. GAPDH was used as internal control. mRNA expression levels were quantified by Image J software and presented as bar diagram (lower panel). **D**. BITC inhibits STAT-3 DNA binding activity in HUVECs. HUVECs were treated with BITC and nuclear fraction was collected. Around 5 µg of nuclear protein subjected to STAT-3 DNA binding activity by Universal EZ-TFA transcription factor assay colorimetric kit according to the manufacturer's protocol. #p<0.01 statistically significant when compared with controls. **E**. BITC inhibits invasion of HUVECs. Invasion assay was performed using Boyden's chamber. *p<0.01 statistically significant when compared with controls. #p<0.01 statistically significant when compared with VEGF-stimulated controls

In a tumor microenvironment, tumor cells secrete VEGF, which promotes the growth of neighboring endothelial cells. Moreover, the complex process of angiogenesis involves the ordered proliferation, assembly, and alignment of endothelial cells [28]. Since we observed that BITC can block VEGF secretion, we next wanted to see if BITC treatment suppresses the secretion of angiogenic proteins from human umbilical vein endothelial cells (HUVEC) through a mechanism similar to what we observed in BxPC-3 and PanC-1 cells. Our results demonstrate that secretion of both VEGF and MMP-2 by HUVECs were significantly inhibited by BITC in a dose-dependent manner (Fig. 5A). Furthermore, we found that BITC down-regulates the expression (Fig. 5B) as well as mRNA levels (Fig. 5C) of MMP-2 and VEGFR-2 in a dose-dependent manner. Consistent with our observations in BxPC-3 and PanC-1 cells, BITC treatment significantly increased the expression of RhoB and decreased the expression of VEGF, RhoA, RhoC, Cdc-42, and Rac1, 2, 3 in HUVECs (Fig 5B). Interestingly, phosphorylation and DNA binding activity of STAT-3 was significantly reduced in BITC treated HUVECs (Fig. 5B & 5D) as compared to control cells. These results suggest that BITC can suppress the angiogenic properties of endothelial cells by inhibiting the expression of proangiogenic proteins. Furthermore, we explored whether BITC inhibits the migration of HUVECs. Our results show that BITC treatment significantly blocked the migration/invasion of VEGF-stimulated HUVECs in a dose-dependent manner (Fig. 5E).

### BITC inhibits *in vivo* pancreatic tumor angiogenesis and tumor growth

**Figure 6:**
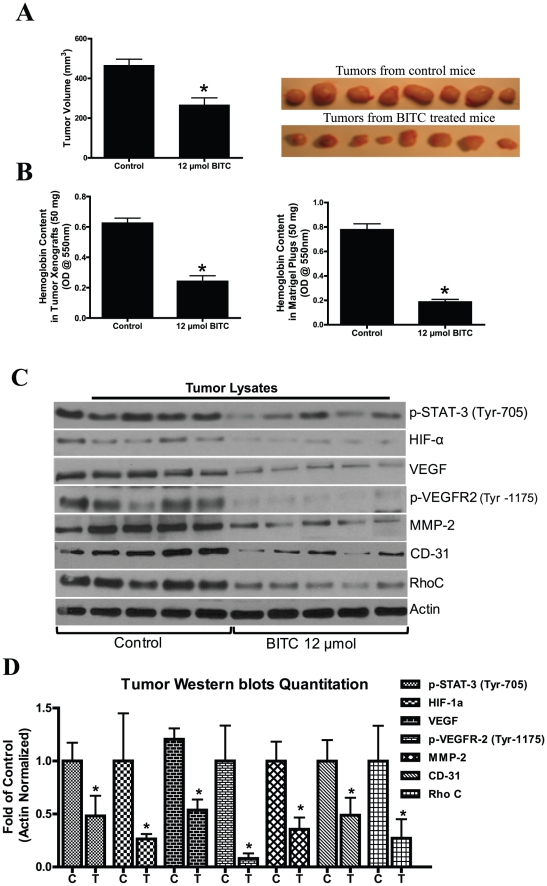
BITC inhibits *in vivo* tumor growth by inhibiting proangiogenic proteins. **A**. BITC suppresses tumor growth *in vivo*. BxPC-3 xenografts bearing mice (n = 10) were orally fed with 12 µmol BITC daily for 40 days. Right side panel shows photographs of isolated tumors from control and BITC-treated mice. **B**. BITC inhibits tumor angiogenesis. BxPC-3 xenografts or matrigel plug-bearing mice were fed with 12 µmol BITC daily for 40 or 7 days, respectively. Tumors and plugs were collected and 50 mg of tumor or plugs were analyzed for hemoglobin content by Drabkin's reagent. **C**. BITC down-regulates pro-angiogenic proteins in tumor xenografts. Cell lysates were prepared from isolated tumor xenografts, subjected to western blot and analyzed for VEGFR-2, MMP-2, HIF-α, and Rho-GTPases **D**. Quantitative analysis of tumor western blots. *p<0.01 statistically significant when compared with controls.

To understand whether BITC directly affects tumor angiogenesis, we performed *in vivo* tumor xenograft experiments in female athymic nude mice. Our results demonstrate that animals tolerated BITC well with no symptoms of toxicity such as weight loss or inactivity was found during BITC administration (data not given). Furthermore, the treatment of mice with 12 µmol BITC markedly suppressed BxPC-3 tumor growth as compared to control group mice (Fig. 6A). In a matrigel plug assay, our results show a 76% reduction in hemoglobin content in BITC treated plugs as compared to untreated plugs (Fig. 6B). Similarly, BITC-treated tumor xenografts showed 61% reduced hemoglobin content as compared to untreated xenografts (Fig. 6B). To further explore the mechanism, tumors were analyzed by western blotting. We observed that phosphorylation of STAT-3 (Tyr-705), VEGR-2 (Tyr-1175) and expression of HIF-α, VEGF, MMP-2, CD31, and RhoC decreased significantly in the tumors of BITC-treated mice as compared to the tumors from control mice (Fig. 6C). Taken together, our *in vivo* results complement our *in vitro* results and clearly indicate the antiangiogenic and antimetastatic potentials of BITC.

## Discussion

Once a tumor reaches a certain size, simple diffusion of oxygen and nutrients to the growing tumor becomes inadequate; hence, specialized blood vessel formation (angiogenesis) is required to meet the increasing demand of nutrition [10]. Angiogenesis is a complex multi-step process that involves cell proliferation, migration, and tube formation. Inhibition of new blood vessel formation leads to a reduced supply of oxygen and nutrients to the tumor, thereby limiting tumor growth (30). Since the growth of tumors is dependent on angiogenesis, its inhibition presents a novel approach to suppressing the growth of solid tumors, including pancreatic tumors [29]. Our results show that sprouting of aortic rings as well as new blood vessel formation on CAM was significantly inhibited by BITC even at sub-lethal concentrations, providing a critical clue to the ability of BITC to inhibit angiogenesis. Furthermore, BITC significantly inhibits the invasion and migration of BxPC-3 and PanC-1 cells, both of which are considered crucial steps in metastasis [8].

In tumor microenvironment, hypoxia stimulates accumulation of HIF-1α protein by decreasing proteasomal degradation. VEGF and MMP-2 involved in angiogenesis are up-regulated by HIF-1α [10], [30]. Previous reports have shown that inhibition of HIF-1α expression leads to decreased tumor growth [31], [32]. Our current results demonstrate that BITC significantly reduces HIF-α accumulation in hypoxia-induced BxPC-3 and PanC-1 pancreatic cancer cells.

Matrix metalloproteinases (MMPs) are capable of degrading numerous matrix components. Several different MMPs are produced in the tumor microenvironment and are strongly implicated in the capillary-sprouting process. Amongst other MMPs, MMP-2 plays a major role in basement membrane degradation [33], [34]. Moreover, MMP-2 production is stimulated by VEGF [35]. Our results reveal that BITC treatment inhibits hypoxia-stimulated secretion of VEGF and MMP-2 in both BxPC-3 and PanC-1 cells. Moreover, BITC also reduces the constitutive secretion of VEGF and MMP-2, which was independent of HIF-1α, in normoxic BxPC-3 and PanC-1 cells. These observations suggest that BITC inhibits tumor cell-secreted proangiogenic factors by both HIF-α dependent and independent mechanism.

VEGF initially was thought to mediate its signaling in a paracrine manner by acting on neighboring endothelial cells, but later it was found that it also acts in an autocrine manner to promote the growth of tumor cells. Various tumors, including pancreatic tumors, are known to express VEGF receptor-2 (VEGFR-2). Further, VEGF binds to VEGFR-1 and VEGFR-2 on endothelial cells and stimulates signaling related to angiogenesis [25]. Our results demonstrate that BITC significantly inhibits phosphorylation (Tyr-1175) and expression levels of VEGF and VEGFR-2 in BxPC-3, PanC-1, and HUVEC cells. Moreover, reduced VEGFR-2 expression was due to decreased transcription of VEGFR-2, as evidenced by reduced mRNA levels in BITC-treated cells. Upon VEGF stimulation, VEGFR-2 undergoes phosphorylation at Tyr-1175, leading to the activation of a number of downstream signaling cascades, including MAPK, PI3K, and PLCγ.

We recently observed that BITC suppresses *in vitro* and *in vivo* pancreatic tumor cell growth by inhibiting the PI3K/AKT pathway [36]. Previous studies have shown that the Rho-family of small GTPases plays an essential role in transmitting the VEGF signals downstream to angiogenesis [37]. Upon VEGF stimulation, c-Src is phosphorylated at Tyr-416 and activated Src interacts with FAK; the Src/FAK complex further activates Rho GTPases [38]. The super-family of Rho-GTPases consists of various Rho-specific insertion domains containing subfamilies such as Rho, Rac, Cdc-42, etc. RhoA and RhoC expression and/or activity frequently is increased in human tumors, whereas RhoB is down-regulated in various cancers [16]. Moreover, increased expression of RhoC correlates with the progression and poor prognosis of ductal adenocarcinoma of the pancreas [39]. The inhibition of Rho-GTPases would lead to decreased metastasis.

Our results clearly demonstrate that BITC treatment significantly reduced the active-RhoA and RhoC protein levels in a dose-dependent manner in both cell lines. RhoA has been implicated in virtually all stages of cancer progression and metastasis, whereas RhoC is restricted to metastasis [40]. Our results provide evidence that both RhoA and RhoC are the targets of BITC in pancreatic cancer cells. Unlike RhoA and RhoC, the level of active-RhoB protein, which is a tumor suppressor gene [41], was up-regulated in both BxPC-3 and PanC-1 cells by BITC treatment. Other Rho-GTPases such as Rac 1, 2, 3 and Cdc-42 are necessary for the generation of lamellipodial protrusions during the mesenchymal migration [42]. BITC treatment significantly reduced the expression levels of Rac 1, 2, 3 and Cdc-42. It is tempting to speculate that BITC may inhibit tumor cell migration by interfering with actin filament rearrangement and/or inhibition of lamellipodial formation. However, further studies are needed to substantiate this assumption.

VEGF-stimulated endothelial cells undergo proliferation, migration, and invasion, leading to new blood vessel formation. Hence, to mimic the tumor microenvironment, we evaluated the effect of BITC on VEGF-treated HUVEC migration, invasion, and secretion of proangiogenic factors. Interestingly, BITC down-regulated the DNA-binding activity and phosphorylation of STAT-3 (Tyr-705), and Rho-GTPases expression in HUVECs, indicating that BITC acts on endothelial cells in a similar fashion as acts on pancreatic epithelial cancer cells. Further, BITC inhibited HUVEC migration, invasion, and VEGF/MMP-2 secretion, indicating that it specifically targets the angiogenic ability of HUVECs.

Previous reports have shown a close association between STAT-3 activation and pancreatic tumor growth and vascularization [14]. Moreover, activation of STAT-3 has been directly correlated with HIF-1α and VEGF (14). Our previous investigations have shown that BITC significantly inhibits the phosphorylation of STAT-3 at Tyr-705 [23]. Hence, we wanted see whether inhibiting the phosphorylation of STAT-3 at Tyr-705 would lead to inhibition of HIF-1α and VEGF expression. In agreement with previously published studies [12], [13], IL-6 treatment significantly increased phosphorylation of STAT-3, HIF-1α and VEGF expression as compared to untreated cells. The effect of BITC on HIF-1α and VEGF expression was significantly diminished in IL-6 treated cells. Previous studies also have shown that phosphorylation of STAT-3 at Tyr-705 is required for dimerization, translocation, and transcriptional activation of HIF-1α [13]. Since IL-6 is a pleiotropic cytokine which elucidates its cellular processes by activating not only STAT-3 but also other targets such as MAPK [43], the association of HIF-1α and VEGF with STAT-3 in our model was further confirmed by ectopic expression of STAT-3. As also shown in previous studies, STAT-3 over-expressing BxPC-3 cells showed a much higher expression of HIF-1α and VEGF as compared to cells expressing constitutive levels of STAT-3 under hypoxic conditions [13]. Hypoxia failed to induce HIF-1α and VEGF expression in STAT-3 silenced or stably knocked out (KO) BxPC-3 cells, indicating that STAT-3 is indispensible for hypoxia-induced expression of HIF-1α and VEGF. When STAT-3 over-expressing BxPC-3 cells were treated with BITC, hypoxia-induced HIF-1α and VEGF expression were significantly diminished; whereas, in STAT-3 KO or transiently silenced cells, BITC caused no change in the expression of HIF-1α or VEGF, indicating that BITC-mediated down-regulation of HIF-1α and VEGF was in fact mediated through STAT-3.

To evaluate the anti-angiogenic activity of BITC *in vivo*, athymic nude mice were injected with BxPC-3 cells and orally gavaged with 12 µmol BITC. BITC suppressed the growth of pancreatic tumor xenografts and also drastically reduced the hemoglobin content in BITC-treated xenografts or implanted Matrigel plugs. These results suggest that BITC inhibits tumor growth *in vivo* not only by directly inhibiting tumor cell proliferation but also through inhibiting tumor angiogenesis. Our results clearly demonstrate a reduced phosphorylation of STAT-3 (Tyr-705), VEGFR-2 (Tyr-1175), and expression of MMP-2, HIF-α, and Rho-GTPases in the tumors of BITC-treated mice as compared to the tumors of control mice. Taken together, our data provides convincing evidence that BITC inhibits pancreatic tumor angiogenesis by targeting STAT-3 and inhibiting HIF-1α/VEGF/MMP-2/Rho-GTPases.

## Materials and Methods

### Cell cultures

Human pancreatic cancer cell lines BxPC-3 and PanC-1 were procured from ATCC (Manassas, VA). BxPC-3 cells have wild type KRas, whereas PanC-1 cells have mutated KRas (G12D). PanC-1 cells are more metastatic as compared to BxPC-3 cells. Both cell lines were maintained as described by us previously [23]. All the antibodies were procured from Cell Signaling Technology Inc. (Danvers, MA), except HIF-α which was obtained from Abcam (Cambridge, MA).

### Aortic sprouting in matrigel

Aortic spouting assay was performed as previously described [44]. In brief, 1 mm long rings were excised from rat thoracic aorta. The rings were submerged in 350 µL Matrigel (BD Biosciences, Bedford, MA) containing 20 ng/mL VEGF. After 24-hour incubation, BITC was added to the rings and incubated for an additional 3–5 days. The aortic rings formed microvascular-like sprouts which were photographed under a light microscope (Olympus Inc., Center Valley, PA). The results were quantified by Image J 1.43 software provided by National Institutes of Health (NIH).

### Chicken chorioallantoic membrane (CAM) assay

The CAM assay was performed according to the method described by Tournaire, et al. [45]. Fertilized chicken eggs were obtained from Charles River Laboratories (Wilmington, MA) and kept at room temperature for 48 hours. On the 4^th^ day at a temperature of 37°C, a 1.5–2 cm window was opened aseptically on each egg shell, exposing the part of the CAM which contained the central vein. The windows were then sealed with sterile Parafilm and the eggs were returned to the incubator for 24 hours. On day 6 of incubation, a Whatman filter paper disc containing 5 µmol BITC was placed in direct contact with the CAM. The windows were again sealed and returned to the incubator. On day 9-12, the seals were removed and neovasculature growth on CAM was photographed with a digital camera (Nikon Inc, Melville, NY).

### Estimation of MMP-2 and VEGF secretion by ELISA

Secreted MMP-2 and VEGF levels in BITC treated BxPC-3 culture medium were measured using an ELISA kit (Invitrogen Corp., Carlsbad, CA) according to manufacturer instructions.

### Reverse transcription-polymerase chain reaction (RT-PCR)

Total RNA was extracted from control and treated cells using TRIzol reagent (Life Technologies, Inc., Carlsbad, CA) according to manufacturer instructions. Amplification was performed using Verso 1-step RT-PCR kit (Thermo Scientific, Surrey, UK) with MMP-2 sense and antisense primers 5′-GTG CTG AAG GAC ACA CTA AAG AAG A-3′ and 5′-TTG CCA TCC TTC TCA AAG TTG TAG G-3′, respectively. VEGFR-2 primers were procured from R&D Systems, Inc. (Minneapolis, MN). PCR amplification consisted of 35 cycles with denaturation at 94°C for 1 minute, annealing at 58°C for 1 minute and extension at 72°C for 1 minute, and a final elongation at 72°C for 10 minutes. The PCR products were separated on a 1.5% agarose gel, stained with 0.5 mg/mL ethidium bromide, and visualized under UV light.

### STAT-3 DNA binding activity

Cells were treated with various concentrations of BITC for 24 h and nuclear fraction was isolated. Around 5 µg of nuclear protein was subjected to STAT-3 DNA binding activity using Universal EZ-TFA transcription factor assay colorimetric kit (Upstate Biotechnology, Inc, Lake Placid, NY) as described by us earlier [22].

### Induction of STAT-3 mediated HIF-1α expression by IL-6 treatment

STAT-3-mediated HIF-1α expression was induced as described earlier, with minor modifications [13]. Briefly, 0.3×10^6^ BxPC-3 cells were plated in a 6-well plate overnight and treated with or without 10 µM BITC in the presence or absence of 20 ng/mL IL-6 for 24 hours. Cells were collected, lysed, and analyzed by western blotting.

### Over expression or silencing of STAT-3

Plasmid containing STAT-3α was transfected in BxPC-3 cells to over-express STAT-3 as previously described [23]. To silence STAT-3, BxPC-3 cells pre-incubated in Opti-MEM medium were transfected with 1.5 µg/ml of STAT-3 shRNA using SiPORT transfection reagent (Ambion, Inc, Carlsbad, CA). After 48 hours of transfection, cells were treated with or without 10 µM BITC for 24 hours under hypoxic conditions.

### Generation of STAT-3 knock out BxPC-3 cells

STAT-3 was stably silenced in BxPC-3 cells using a shRNA kit from SA Biosciences (Frederick, MD). Briefly, BxPC-3 cells were transfected with STAT-3 shRNA using lipofectamine and allowed to grow according to manufacturer's instructions. Stable selections were made in the presence of G418. Surviving transfected cells were grown in the presence of 100 µg/ml G418 for at least five passages before using them for any experiment. We were able to silence about 85% of STAT-3 expression in these cells.

### Wound healing and transwell migration assay

Wound healing assay was performed as described earlier [46]. Confluent monolayers of BxPC-3 cells in 6-multiwell plates were scratched with a 1 mL pipette tip and incubated in RPMI medium containing 5 µM BITC. Cells were photographed under a microscope (Olympus America, Inc, Center Valley, PA) at the intervals of 0, 12, 24, 36 and 48 hours. Results were quantified by Image J 1.43 software (NIH). Cell invasion was performed in Transwell Boyden chamber containing 8.0 µm pore size filters (BD Biosciences, Bedford, MA). Briefly, cells were serum starved overnight and harvested by tripsinization. A suspension of 20,000 cells in RPMI medium (600 µL) containing 1% serum were seeded on the upper well of Boyden chamber while lower chamber was filled with 1.5 mL of media with 1% serum. After incubation for 6 h, cells were pre-treated with 10 µM mitomycin C for 1 h and then BITC was added to upper chamber whereas, 10% FBS and 20 ng/mL HGF/VEGF was added to lower chamber as a chemo-attractant. After incubation for 24 h, cells on the upper surface were removed by wiping with a cotton swab. The filters containing the cells were removed from the Transwell chambers and individually transferred to separate wells in a 96-well plate and developed as described by the manufacturer.

### Western blot analysis

Treated and untreated BxPC-3/PanC-1 cells were washed twice with PBS, lysed, and subjected to western blot analysis as previously described by us [22].

### *In Vivo* tumor xenograft assay

All the experiments involving animals were approved by the Institutional Animal Care and Use Committee (IACUC), Texas Tech University Health Sciences Center and the experiments were conducted in strict compliance and regulations. Twenty 4–8 week old female athymic nude mice (Charles River, Wilmington, MA) were kept on an antioxidant-free AIN-76A diet (TestDiet, Richmond, IN) for 1 week before starting the experiment. About 1×10^6^ exponentially growing BxPC-3 cells were injected subcutaneously into the left and right flanks so that each mouse had two xenografts. Tumor volumes were measured three times a week [22]. Once each mouse had palpable tumors, mice were randomly separated into two groups of 10 mice each. Mice in the experimental group received 12 µmol BITC by oral gavage every day, whereas the control mice received vehicle alone. At the end of the experiment, mice were sacrificed humanely by in accordance with IACUC guidelines; tumors were excised from each mouse and snap frozen.

### Matrigel plug assay

Matrigel plug assay was performed as described earlier [44]. A Matrigel (BD Biosciences, Bedford, MA) mixture (250 µL) containing 40 ng/ml VEGF was injected subcutaneously into 4–8-week-old female athymic nude mice (n = 10) on both flanks. The mice were daily gavaged with 12 µmol BITC for 7 days, after which they were euthanized and the matrigel plugs were recovered. The plugs were dispersed in PBS and incubated at 37°C overnight. Hemoglobin levels were determined using Drabkin's reagent (Sigma-Aldrich, St. Louis, MO) according to manufacturer instructions.

### Hypoxia treatment

For hypoxia treatment, cells were treated with various concentrations of BITC for 24 hours and then exposed to hypoxic conditions (1% O_2_) for 12 hours in drug-free hypoxic media. The medium was assayed for MMP-2 and VEGF and the cells were subjected to western blot analysis.

### Statistical analysis

All the statistical analyses were performed using Prism 5.0 (GraphPad Software Inc., San Diego, CA). Results were expressed as mean ± SD or S.E.M. for at least three independent experiments. Data were analyzed by Student's *t*-test or one way ANOVA followed by Bonferroni's post-hoc analysis for multiple comparisons. Differences were considered statistically significant at p<0.05.
